# Comprehensive ABA-based interventions in the treatment of children with autism spectrum disorder – a meta-analysis

**DOI:** 10.1186/s12888-022-04412-1

**Published:** 2023-03-02

**Authors:** Theresa Eckes, Ulrike Buhlmann, Heinz-Dieter Holling, Anne Möllmann

**Affiliations:** 1 Institute of Psychology, University of Münster, Fliednerstr. 21, 48149 Münster, Germany; 2 Institute of Psychology, Clinical Psychology and Psychotherapy, University of Bremen, Grazer Str. 6, 28359 Bremen, Germany

**Keywords:** Autism Spectrum Disorder, Applied Behavior Analysis, Behavioral Treatment, Early Intensive Behavioral Interventions, Meta-Analysis, Parental Stress

## Abstract

Many studies display promising results for interventions that are based on Applied Behavior Analysis (ABA) in the treatment of autism spectrum disorder (ASD). Methods: This meta-analysis assessed the effects of such treatments on developmental outcomes in children with ASD and on parental stress based on 11 studies with 632 participants. Results: Compared to treatment as usual, minimal or no treatment, comprehensive ABA-based interventions showed medium effects for intellectual functioning (standardized mean difference SMD = 0.51, 95% CI [0.09; 0.92]) and adaptive behavior (SMD = 0.37, 95% CI [0.03; 0.70]). Language abilities, symptom severity or parental stress did not improve beyond the improvement in control groups. Moderator analyses indicate that language abilities at intake could influence the effect sizes and the influence of treatment intensity might decrease with older age. Conclusions: Practical implications and limitations are discussed.

**Supplementary Information:**

The online version contains supplementary material available at 10.1186/s12888-022-04412-1.

Autism spectrum disorder (ASD) is a neurodevelopmental disorder characterized by (a) difficulties in social communication and interaction across multiple contexts and (b) restricted, repetitive behavior, activities, and interests. It is often associated with intellectual impairment, language impairment, and motor deficits, such as odd gait or clumsiness [1]. According to the clinical criteria of ASD laid out in the Diagnostic and Statistical Manual of Mental Disorders (DSM-5; [1]), the prevalence of ASD is approximately 1.0% and people on the autistic spectrum need intensive, sometimes life-long care and support [2].

Behavioral interventions for ASD target the increase of functional independence of individuals on the autistic spectrum. They are firmly linked to Applied Behavior Analysis (ABA) [2, 3]. ABA is the science of analyzing how the individual’s environment influences their behavior [4] and describes interventions applying the findings of such analyses to change behavior [5, 6]. It is theoretically based on operant conditioning and aims to assess and change challenging behavior as well as to promote and generalize more adaptive behavior, for example, by using systematic reinforcement [5]. While ABA-based methods can be used to target specific behaviors (e.g,. toilet training), comprehensive ABA-based interventions are characterized by (a) beginning in early childhood, when possible between 3 to 4 years of age; (b) having a high intensity (20 – 40 h/week); (c) being personalized to meet the individual needs of each child; (d) addressing several skills at the same time instead of promoting just one specific skill (e.g., joint attention); and (e) using multiple behavior analytic methods. Additionally, comprehensive ABA-based interventions (f) use a one-to-one format that is gradually supplemented with group activities and transferred to naturalistic contexts, and (g) require parental participation ([7] as cited in [8]). Well-known examples for comprehensive ABA-based interventions are Early Intensive Behavioral Interventions (EIBI) [4], which make up the majority of the studies considered in the present study. However, since some comprehensive, intensive ABA-based methods are not called EIBI, for instance the Verbal Behavior approach (ABA-VB, for a detailed description see [9]), we will use the term comprehensive ABA-based interventions rather than EIBI in our study. While treatment goals in behavioral interventions are most often in line with typical sequences of development (e.g., the promotion of more adaptive behavior), another group of interventions is explicitly designed on applying behavioral methods (e.g., methods based on ABA) *and* developmentally-based strategies in naturalistic settings, deriving individual learning objectives from developmental sequences [10]. Those interventions are called Naturalistic Developmental Behavioral Interventions (NDBI) [10]. A prominent example for NDBI is the Early Start Denver Model (ESDM). This group of treatment interventions shows good evidence of efficacy in recent meta-analyses [11, 12]. Because of conceptual differences, NBDIs are not included in the present meta-analysis.

Comprehensive ABA-based interventions are widely used in North America in the treatment of ASD. In Europe, however, comprehensive ABA-based programs are rarely applied [13], among other things due to the claim that they are not evidence-based [2]. This claim is rooted in the fact that many studies that investigate comprehensive ABA-based therapies are of poor methodological quality [2, 14]. As Reichow and colleagues [14] showed in their meta-analysis, many studies investigating the effects of EIBI in autistic children have small samples, a non-optimal design and a high risk of bias according to the GRADE system. Additionally, other reasons like financial or cultural obstacles when implementing comprehensive ABA-based treatments are discussed [13]. It should not be neglected that some aspects (e.g., the intensive use of reinforcement) of comprehensive ABA-based treatments raised ethical concerns about this approach [13, 15]. However, comprehensive ABA-based interventions, like EIBI, provide a substantiated theoretical basis.

Nine meta-analyses on the effects of comprehensive ABA-based interventions on intellectual functioning, adaptive behavior (e.g., communication skills and socialization) and language abilities were published between 2009 and 2018 [8, 16–23]. Eight meta-analyses found comprehensive ABA-based interventions to be more effective in the treatment of children with ASD than standard care [8, 16, 1, 18–22]. Solely Spreckley and Boyd [22] concluded that the interventions are not superior to standard care.

The meta-analyses have some methodological problems, such as a risk for biased effect sizes, the inclusion of studies without an appropriate control group or the use of fixed-effect models. Specifically, the use of uncontrolled pre-post-comparisons to calculate an effect size (as used in [8, 17, 18, 21]) is susceptible for threats of validity and may lead to overestimation of the effect size [16]. Effect sizes can also be biased, if they are not standardized (as seen in [21]). The use of fixed-effect models while including studies with more than one control group (e.g., [24]) is problematic because these models do not control for the dependence of effect sizes (as seen in [16]). Finally, an underestimation of effect sizes can occur from primary studies that compare two intensive ABA-based treatments, like the study from Sallows and Graupner [25] or the study by Smith, Groen and Wynn [26] (as seen in [14, 19, 22]). Considering the limitations of previous meta-analyses, applying a more rigorous meta-analytic methodology appears warranted.

Adding to this, the methodological quality of many primary studies investigating interventions for autistic children is low [14, 27]. This could result in inflated effect sizes and, thus, bias the meta-analytic conclusions drawn from those studies. Some (e.g., [14, 19, 27]) but not all previous meta-analyses have considered the risk of bias of primary studies in order to assess the certainty of the results. However, this procedure is essential when conducting a meta-analysis in a research field with many primary studies that are limited in their methodological quality.

Further, several studies (primary studies and meta-analyses) have pointed towards potential factors, that might moderate the impact of comprehensive ABA-based treatments on developmental outcomes. Potential moderators are especially relevant for the improvement and personalization of treatment methods. Possible moderators are higher intellectual functioning (e.g., [28]), higher language abilities [27], more adaptive behavior, and less severe psychopathology at intake [28–31]. Some studies discuss age as a potential moderator of the effectiveness of comprehensive ABA-based interventions (e.g., [27, 29–32]). Furthermore, treatment intensity and duration, *cumulative intervention intensity* as well as parental training and participation (as therapists) could impact the outcome [17, 20, 27, 31–34]. A recent meta-analysis on the effects of early interventions on social communication in autistic children [35] showed that interventions with parental participation had slightly smaller effect sizes than interventions provided by clinicians only. This result contrasts earlier results [36] in which interventions provided by parents and clinicians seemed to improve the effect of treatment compared to parent-only or clinician-only interventions. Further, there is evidence that maternal involvement in comprehensive ABA-based treatments is connected to mothers’ personal strain [37]. That is, the necessity of parental participation is unclear and might be associated with negative consequences for the parents. Overall, findings from meta-analyses [8, 17, 20] as well as findings in primary literature regarding potential moderators are heterogeneous and thus rather inconclusive. For example, Makrygianni and Reed [18] showed that treatment intensity is correlated with treatment gains in intellectual functioning and adaptive behavior, whereas Reichow and Wolery [20] did not find evidence for any impact of treatment intensity or duration.

In the current meta-analysis, we first aim to replicate findings of previous studies regarding impacts of comprehensive ABA-based interventions on adaptive behavior, intellectual functioning, language abilities, and symptom severity, applying a more rigorous methodology. Based on the results of preceding meta-analyses, we assume that comprehensive ABA-based (vs. control group) interventions improve adaptive behavior, intellectual functioning, language abilities (expression and comprehension), and symptom severity.

Second, we aim to investigate possible moderators of treatment outcomes. We hypothesize that comprehensive ABA-based interventions are more effective for younger children (e.g., [32]), with fewer impairments in adaptive behavior (e.g., [28]), intellectual functioning (e.g., [28]), language abilities (e.g., [31]), and with lower symptom severity (e.g., [30]) at intake. Additionally, we assume that parental participation (e.g., [36]), longer treatment duration (e.g., [34]), higher treatment intensity (e.g., [17]), and higher cumulative intervention intensity (e.g., [32]) increase the effectiveness of comprehensive ABA-based interventions. Finally, we hypothesize that the impact of treatment duration and (cumulative) intensity is higher in younger children (based on the findings of [32]).

Third, we aim to investigate whether this kind of intervention has an impact on parental stress. Parents of children with ASD experience greater stress than parents of typically developing children or children with other disabilities [38]. Thus, we hypothesize that comprehensive ABA-based interventions might reduce parental stress by reducing children’s symptom severity. But, as mentioned above, involvement in comprehensive ABA-based interventions can decrease parental well-being [37]. Accordingly, parental stress might be increased due to the high demands of comprehensive ABA-based interventions (for example, delivering treatment to the child in “almost all of the subjects' waking hours, 365 days a year” [39]).

## Method

### Eligibility criteria

Studies included in this meta-analysis had to meet the following criteria: 1) ASD was diagnosed according to the International Statistical Classification of Diseases and Related Health Problems (ICD 10) or DSM IV criteria; 2) studies provided a control group (i.e. randomized controlled trials (RCTs), quasi-randomized trials and controlled clinical trials); 3) treatment groups had at least five participants; 4) at least one group had to receive a comprehensive ABA-based intervention, as defined previously, for more than 10 h per week^1^; 5) control groups received treatment as usual (TAU) or an alternative active intervention (no comprehensive ABA-based intervention with more than 10 h of treatment per week); 6) at least one child-related outcome (adaptive behavior, intellectual functioning, language abilities or symptom severity) was reported; 7) mean and standard deviation for each outcome were reported, computable or provided by the authors of the study; 8) the study was published in English or German in a peer-reviewed journal or as part of a doctoral dissertation. We did not include retrospective or epidemiological studies, merely qualitative studies or studies without standardized outcome measures. Additionally, although many studies in the field of comprehensive ABA-based interventions for autistic children are single case experimental studies [e.g., 12], we decided to only include studies with a controlled design in order to reduce heterogeneity of included studies and, thus, promoting the validity of the integrated results.

### Search methods for identification of studies

We conducted a literature search in the databases Medline, Psyndex, PsycInfo, and PsycArticles from January 1 2018 until March 6 2018 and updated this search from March 5 2020 until March 9 2020. Additionally, we searched Google Scholar and considered relevant studies from reference lists of preceding meta-analyses [8, 15–22]. We did not restrict study obtainment by publishing date. We used following search terms (English and German equivalents): *ASD*, *autism*, or *autism spectrum disorder* AND *EIBI*, *ABA*, *early intensive behavio(u)r intervention*, *applied behavio(u)r analysis*, *comprehensive ABA*, *early intensive behavio(u)r treatment*, *UCLA-model*, *Lovaas*, *intensive or behavio(u)r training*. Our search term also included *Early Start Denver Model* and *pivotal response training* to broaden the results of our literature search. However, studies that only focused on those (NDBI) interventions were not included in our analysis. The full search strategy is listed in the supplementary material A.

### Data collection and quality assessment

Title and abstract of all distinct reports were screened, and all potentially applicable studies were coded. A second independent rater assessed and coded nine (12%) of the 75 potentially applicable studies. The inter-rater agreement for eligibility was low (Cohen’s κ = 0.4), so discrepancies were discussed among the authors and studies in question were reassessed. Additionally, eight more studies were assessed by the second rater, so that in total 22% of studies were rated by two independent raters. After discussion, reassessment and additional coding, inter-rater agreement for study eligibility reached Cohen’s κ = 1.0. Both raters used a data collecting form (supplementary material A). We calculated inter-rater agreement before and after the discussion for all relevant outcomes and moderators (see supplementary material B). Please note, that the moderators were only rated after discussion and reassessment.

To assess risk of bias for each study, we used the “Cochrane Collaboration’s tool for assessing risk of bias” [23]. A brief description of this tool can be seen in supplementary material C.

### Statistical analyses

We calculated standardized mean differences (SMDs) between groups as effect size for each outcome as recommended by Viechtbauer [40] for continuous data and measures with different scales and corrected SMDs for bias resulting from small sample sizes [41]. As recommended by the Cochrane handbook of systematic reviews [23], we used post-treatment comparisons in the effect size calculation. Table 1 provides a summary of formulas used in this meta-analysis.

**Table 1:** Formulas used in the meta-analysis adapted from Borenstein et al. (2009)

Statistic	Formula
Standardized Mean Difference^1^	$\mathbf{S}\mathbf{M}\mathbf{D}=\frac{\left({{\varvec{M}}}_{\mathbf{T}\mathbf{p}\mathbf{o}\mathbf{s}\mathbf{t}}-\boldsymbol{ }{{\varvec{M}}}_{\mathbf{C}\mathbf{p}\mathbf{o}\mathbf{s}\mathbf{t}}\right)}{{{\varvec{S}}{\varvec{D}}}_{\mathbf{p}\mathbf{o}\mathbf{o}\mathbf{l}\mathbf{e}\mathbf{d}}}\times \left(1-\frac{3}{4\times {\varvec{d}}{\varvec{f}}-1}\right)$
Pooled Standard Deviation^1^	${{\varvec{S}}{\varvec{D}}}_{\mathbf{p}\mathbf{o}\mathbf{o}\mathbf{l}\mathbf{e}\mathbf{d}}=\sqrt{\frac{\left({{\varvec{n}}}_{\mathbf{T}}-1\right){{\varvec{S}}{\varvec{D}}}_{\mathbf{T}\mathbf{p}\mathbf{o}\mathbf{s}\mathbf{t}}^{2}+\left({{\varvec{n}}}_{\mathbf{C}}-1\right){{\varvec{S}}{\varvec{D}}}_{\mathbf{C}\mathbf{p}\mathbf{o}\mathbf{s}\mathbf{t}}^{2}}{{{\varvec{n}}}_{\mathbf{T}}+{{\varvec{n}}}_{\mathbf{C}}-2}}$
Variance of SMD	${{\varvec{V}}}_{\mathbf{S}\mathbf{M}\mathbf{D}}={\left(1-\frac{3}{4\times {\varvec{d}}{\varvec{f}}-1}\right)}^{2}\boldsymbol{ }\times \boldsymbol{ }\left(\frac{{{\varvec{n}}}_{\mathbf{T}}+\boldsymbol{ }{{\varvec{n}}}_{{\varvec{C}}}}{{{\varvec{n}}}_{\mathbf{T}}{{\varvec{n}}}_{\mathbf{C}}}+\frac{\mathbf{S}\mathbf{M}{\mathbf{D}}^{2}}{2\boldsymbol{ }\times \left({{\varvec{n}}}_{\mathbf{T}}+\boldsymbol{ }{{\varvec{n}}}_{{\varvec{C}}}\right)}\right)$
Standard Error of SMD	${{\varvec{S}}{\varvec{E}}}_{\mathbf{S}\mathbf{M}\mathbf{D}}=\boldsymbol{ }\sqrt{{{\varvec{V}}}_{\mathbf{S}\mathbf{M}\mathbf{D}}}$

*Note.*^1^ SMD and SD_pooled_ for post-treatment comparisons. SMD = standardized mean difference; *V* = variance; *SE* = standard error; *M* = mean; *SD* = standard deviation; *n* = sample size; *df* = degrees of freedom; T = treatment group; C = control group; pre = pre-treatment measurement; post = post-treatment measurement

In studies with multiple intervention groups, we included the comparison between the two most relevant intervention groups only to prevent an uneven weighting of the sample used in those comparisons. We investigated heterogeneity of the included studies by computing *Q* statistics, assessing variation of the true effect sizes between studies through ${\upsigma }_{B}^{2}$, and calculating *I*^2^. We evaluated the risk for publication bias by a visual inspection and a test of funnel plot asymmetry, according to Egger and colleagues [42]. We conducted this test only for outcomes with more than 10 effect sizes and with univariate models, as recommended by Sterne and colleagues [43].

We calculated a random effects model because we assumed varying true effect sizes due to differences between specific treatments. To address the fact that most outcomes were measured in different dimensions, we used a multilevel meta-analysis model. We computed this meta-analysis using the R-package “metafor” (package version: 1.9–4, R-studio version: 1.1.447) [40, 44].

We conducted moderator analyses with children’s age and abilities (intellectual functioning, adaptive behavior, language before treatment), treatment intensity, and duration. Further, we computed an additional variable called *cumulative intervention intensity* (hours/week × 4,33 × duration in months). Because the number of studies reporting symptom severity at intake was too low and treatment was delivered by parents and therapists in all studies, we had to drop the moderators symptom severity and parental participation from the analyses.

## Results

### Study and sample characteristics

**Fig. 1:**
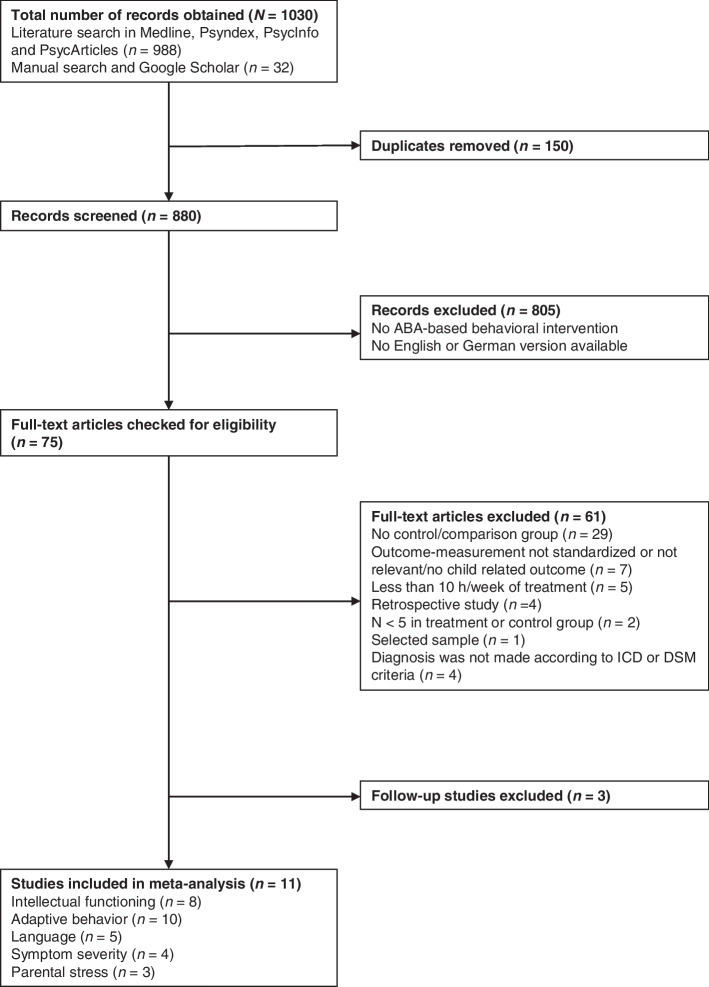
Flow chart of study obtainment process

Fourteen studies from the initial literature search met the eligibility criteria. Two of those studies were excluded because they assessed follow-up samples of other included studies [45, 46]. Thereby we avoided including the same sample multiple times. Furthermore, we were not able to obtain the pre-treatment measurement for one study [47]. The update of the literature search in 2020 did not reveal additional eligible studies. Thus, we included 11 studies with 632 participants in our meta-analysis. Figure 1 illustrates the study selection  process.

Most included studies were clinical controlled trials with a quasi-experimental design. Only the study from Shawler [48] was a RCT. As Table 2 shows, eight out of 11 studies, compared comprehensive ABA based treatments to treatment as usual (TAU). Specifically, TAU contained eclectic treatment strategies combining a variety of interventions mostly from Treatment and Education of Autistic and Related Communication Handicapped Children principles (TEACCH principles, [58]), Picture Exchange Communication System (PECS, [59]), ABA-based interventions, Makaton [60], and speech and occupational therapies. No study provided a quantification of the extent to which each intervention was applied. One study [24] included two control groups. ﻿We﻿ only considered one control group, specifically the eclectic educational program, which was more similar to the TAU control groups.

**Table 2:** Study and participant characteristics

Author (Year), Country	N (T/C)	Age (T/C)	Male (%)	Control group	Intensity T/C (h/week)	Duration T/C (months)	Outcome (Instrument)
Eikeseth et al. (2002), Norway [49]	13/ 12	66.32/ 65	76	TAU	28/29.8	12/12	IF (WPPSI-R, WISC-R/-III, BSID-II), AB (VABS), L (RDLS)
Eikeseth et al. (2012), Norway [50]	35/ 24	47/ 53	83.1	TAU	23/NA	12/12	AB (VABS)
Eldevik et al. (2012), Norway [51]	31/ 12	42.2/ 46.2	76.7	TAU	13.6/NA	25.1/24.6	IF(BSID,SB:FE), AB (VABS)
Fava et al. (2011), Italy [52]	12/ 10	52/ 43.7	86.4	TAU	14/12	6.4/7.2	IF (GMDS-ER 2:8), AB (VABS), L (CDI), SyS (ADOS), PS (PSI)
Fernell et al. (2011), Sweden [53]	91/ 101	37.6/ 43.5	NA	low-intensity treatment (ABA)	15–40/NA	25/NA	AB (VABS)
Howard et al. (2005), USA [24]	29/ 16/16	30.86/ 37.44/ 34.56	88.5	(1) AP (2) GP	25–40/25–30	14.12/13.25	IF (BSID-II^a^), AB (VABS^a^), L (RDLS^a^)
Magiati et al. (2007), UK [54]	28/ 16	38/ 42.5	88.6	TAU	32.4/25.6	24/26	IF (BSID-II, WPPSI-R), AB (VABS), L (BPVS-II, EOWPVT-R), SyS (ADI-R)
Molnár et al. (2017), Germany [55]	13/ 7	56.4/ 56.4	100	waitlist control group	17.5/0	10.7/6	IF (PEP-3)
Remington et al. (2007), UK [56]	23/ 21	35.7/ 38.4	NA	TAU	25.6/NA	24/24	IF (BSID-II, SB:FE) AB (VABS), L (RDLS-3), SyS (ASQ), PS (QRS-F)
Shawler (2016), USA [48]	32/ 19	27.97/ 27.24	86.3	TAU	22/1–8 (h/ month)	13.16/13.05	IF (MSEL)
Zachor & Ben-Itzchak (2010), Israel [57]	45/ 33	25.1/ 26	91	TAU	20/19	12/12	AB (VABS), L (MSEL)

*Note.*^a^several other instruments were used as described in the section “Methods of outcome measurement”. Age is displayed in months. N = sample size, T = treatment group, C = control group, TAU = treatment as usual (eclectic treatment), AP = Autism educational programming, GP = General educational programming, IF = intellectual functioning, AB = adaptive behavior, L = language abilities, SyS = symptom severity, PS = parental stress, MSEL = Mullen Scales of Early Learning, VABS = Vineland Adaptive Behavior Scale, ADOS = Autism Diagnostic Observation Schedule, WPPSI-R = Wechsler Preschool and Primary Scale of Intelligence, WISC-R/WISC-III = Wechsler Intelligence Scale for Children, BSID = Bayley Scales of Infant Development, RDLS = Reynell Developmental Language Scale, SB:FE = Standford-Binet Intelligence Scale: Fourth Edition, GMDS-ER 2:8 = Griffith Mental Developmental Scales—Extended Revised: 2 to 8 Years, CDI = MacArthur Communication Developmental Inventories, PSI = Parenting Stress Index, BPVS-II = British Picture Vocabulary Scale- II, EOWPVT-R = Expressive One-Word Picture Vocabulary Test-Revised, ADI-R = Autism Diagnostic Interview – Revised, PEP-3 = Psychoeducational Profile, ASQ = Autism Screening Questionnaire, QRS-F = Questionnaire on Resources and Stress–Friedrich short form

One study compared an ABA-based treatment to another active treatment [53]. However, other than the studies by Sallows and Graupner [25] and Smith and colleagues [26], this study did not use intensive, comprehensive ABA-based treatment in the control group, but instead used low-intensity, targeted ABA-based training. Finally, one study used a waitlist control design. On average, comprehensive ABA-based interventions had an intensity of 21.84 h per week (*SD* = 5.90, ranging from 13.6 to 32.4 h/week) and the control group treatments 17.19 h per week (*SD* = 10.83, ranging from 0 to 29.8 h/week). For a detailed study and participant description see Table 2.

### Outcome measures

The outcomes were assessed with many different measures (see supplementary material D for a complete list of all instruments). The most frequently used instruments will be described in the following. *Adaptive behavior* was mostly measured with the Vineland Adaptive Behavior Scale (VABS I or II) [61, 62] on the four scales communication, socialization, daily living, and motor skills. To assess *intellectual functioning,* most studies administered the Mullen Scales of Early Learning (MSEL) [63], the Bayley Scales of Infant Development (BSID) [64, 65], the Stanford-Binet Intelligence Scale: Fourth Edition (SB:FE) [66], the Wechsler Preschool and Primary Scale of Intelligence (WPPSI-R) [67], or the Wechsler Intelligence Scale for Children (WISC-R, WISC-III) [68, 69]*.* The MSEL and BSID mainly examine motor skills, language, and behavioral abilities, the WPPSI-R, SB:FE, and WISC-R rather assess verbal comprehension, reasoning, knowledge, and memory. The Reynell Developmental Language Scale (RDLS, RDLS III) [70, 71] was used to measure *language comprehension and expression* in most studies. *Symptom severity* was assessed by the Autism Diagnostic Observation Schedule (ADOS) [72], the Autism Diagnostic Interview – Revised (ADI-R) [73], or the Autism Screening Questionnaire (ASQ) [74]. ADOS is based on ratings of an assessor, ADI-R is a structured parent interview and the ASQ is a questionnaire for parents. *Parental stress* was measured with the self-report questionnaires Parenting Stress Index (PSI) [75] and the Parent and Family Problems subscale of the Questionnaire on Resources and Stress–Friedrich short form (QRS-F) [76]. Studies that applied more than one measure for a construct, provided a mean value for that construct across all instruments (e.g., on the IQ scale), which was used for the effect size calculation.

### Risk of bias

Table 3 displays an overview of risk of bias in each study.

**Table 3:** Risk of Bias for included studies

Author(s) (Year)	Sequence generation	Allocation sequence concealment	Masking of participants and personnel^a^	Masking of outcome assessment^a^	Incomplete data	Selective reporting	Other risks of bias
Eikeseth et al. (2002) [49]	High risk (availability of personnel)	High risk	High risk	Low risk	Low risk	Low risk	High risk (contamination, baseline imbalance)
Eikeseth et al. (2012) [50]	High risk (type of hospital/center)	High risk	High risk	High risk	High risk	High risk	High risk (contamination)
Eldevik et al. (2012) [51]	High risk (type of hospital/center)	High risk	High risk	Low risk	Unclear risk	Low risk	High risk (contamination)
Fava et al. (2011) [52]	High risk (parental preference)	High risk	High risk	High risk	Unclear risk	Low risk	High risk (contamination)
Fernell et al. (2011) [53]	High risk (post-hoc assignment)	High risk	High risk	High risk	High risk	Low risk	High risk (contamination, baseline imbalance)
Howard et al. (2005) [24]	High risk (Placement by educational team/ parents)	High risk	High risk	Unclear risk	High risk	Low risk	High risk (contamination, baseline imbalance)
Magiati et al. (2007) [54]	High risk (decision was made before study assignment)	High risk	High risk	Unclear risk	Low risk	Low risk	High risk (baseline imbalance)
Molnár et al. (2017) [55]	High risk (date of assignment)	High risk	High risk	High risk	Low risk	Low risk	Unclear risk (baseline imbalance)
Remington et al. (2007) [56]	High risk (parental preference)	High risk	High risk	Low risk	Unclear risk	Low risk	High risk (baseline imbalance)
Shawler (2016) [48]	Unclear risk	Unclear risk	High risk	Unclear risk	Low risk	Low risk	Low risk
Zachor & Ben-Itzchak (2010) [57]	High risk (place of residence)	High risk	High risk	High risk	Unclear risk	Low risk	Low risk

*Note*. ^a^assessed for all outcomes combined, because they would all be affected by performance or detection bias in the same way

### Selection bias (sequence generation and allocation sequence concealment)

One included study used a randomized procedure to assign participants to groups [48]. All other studies used quasi-experimental designs and thus had a higher risk of selection bias. However, Shawler [48] did not provide enough information about the randomization process and allocation concealment, so the real risk of selection bias remains unclear for this study.

### Performance and detection bias (Masking of participants and personnel/ outcome assessment)

Participants (and their parents) as well as personnel were not masked in any study. Outcome assessors were truly masked in one study [49]. In one study [51], only 60% of cases were evaluated by a masked assessor but they controlled for the other 40% and found no evidence of bias. Thus, we labeled this study with a low risk of detection bias.

### Attrition bias (incomplete data)

The risk of incomplete data and therefore of attrition bias was low in four studies [48, 49, 54, 55]. Four studies did not report how many participants were reassessed after the intervention. Therefore, risk of bias is unclear [51, 52, 56, 57]. High risk for attrition bias emerges from four studies. In one study [50], data for the post-treatment-measurement was available for only 25% (adaptive behavior) or 22% (symptom severity) of children. Fernell and colleagues [53] reported that 10 out of 208 children were not assessed after the intervention. Several other children participated only in some of the required outcome measurements. Neither reasons for this lack of participation nor the amount of withdrawals for each group were stated. In the study of Howard and colleagues [24], 22% of participants (17 out of 78) dropped out for unknown reasons. Additionally, some of the remaining children did not complete all outcome measurements.

### Reporting bias (selective reporting)

We did not find selective reporting in most studies. Eikeseth and colleagues [50] reported symptom severity for the treatment group only and did not conduct a between-group comparison for this outcome. Therefore, this study might be affected by reporting bias.

### Other risks of bias

Six studies reported that the control group received or might have received some treatment based on ABA-techniques [24, 49–53]. Furthermore, intervention groups differed substantially at baseline in five studies [24, 49, 53, 54, 56]. In studies by Fernell and colleagues [53], Howard and colleagues [24], and Remington and colleagues [56], children in the treatment group were significantly younger than children in the control group. Magiati and colleagues [24, 54] reported that children in treatment group scored higher in intellectual functioning (83 vs. 62.5 [IQ scale], *p* = 0.04), in the composite score of adaptive behavior (60.3 vs. 56.6 [standard score], *p* = 0.05) and in the socialization subscale of adaptive behavior (59.6 vs. 55.4 [standard score], *p* = 0.04) at intake.

### Effects of intervention

#### Adaptive behavior

**Fig. 2:**
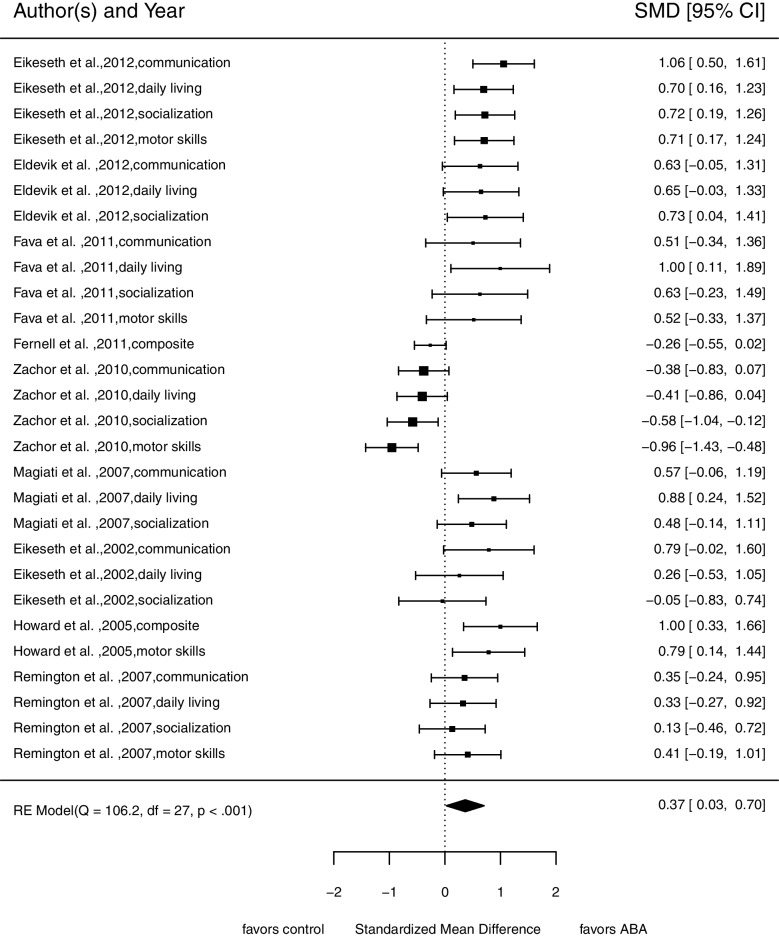
Forrest plot of pooled *SMD* in post-treatment scores in adaptive behavior

For this analysis, we included 28 comparisons from nine studies with 547 participants at pre-treatment measurement. Figure 2 illustrates evidence that comprehensive ABA-based treatments improve adaptive behavior more strongly compared to TAU, minimal or no treatment (SMD = 0.37, 95% confidence interval (CI) [0.03; 0.70]). We found substantial variance between studies (${\upsigma }_{B}^{2}$ = 0.24) but no variance within the studies (${\upsigma }_{W}^{2}$ = 0.00). The tests for heterogeneity indicated substantial heterogeneity (*Q*(*df* = 27) = 106.18, *p* < 0.001; *I*^2^ = 72.95). Statistical testing (*z* = 3.36, *p* < 0.001) and funnel plot inspection (Fig. 7) indicated a publication bias.

### Intellectual functioning

**Fig. 3:**
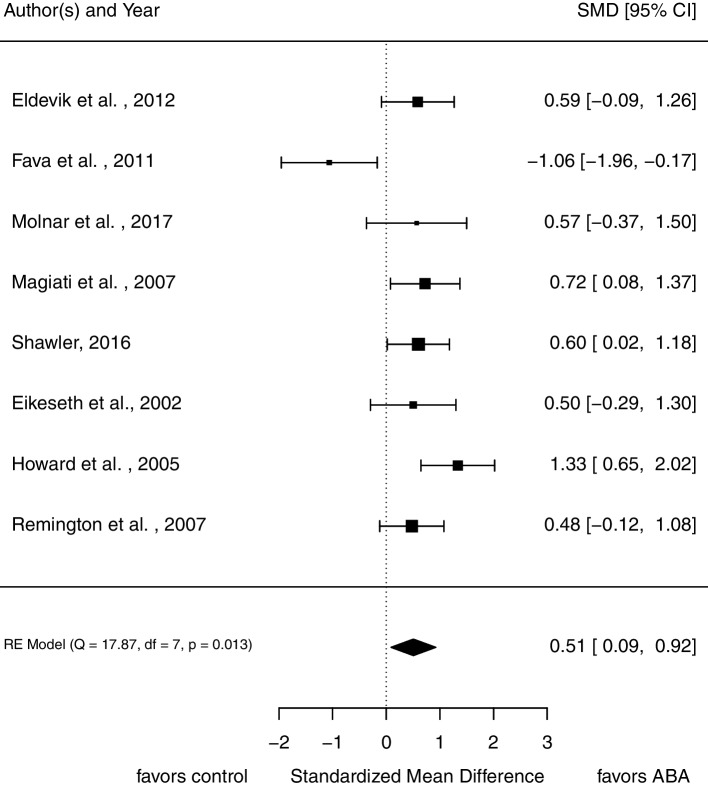
Forrest plot of pooled *SMD* in post-treatment scores in intellectual functioning

Eight studies assessed intellectual functioning (*N* = 293 at pre-treatment assessment). As displayed in Fig. 3, participants in treatment groups show significantly more improvement in intellectual functioning than participants in control groups (*SMD* = 0.51, 95% CI [0.09, 0.92]). There is considerable variance between studies (${\upsigma }_{B}^{2}$ = 0.22). The *Q* statistic, *Q*(7) = 17.87, *p* = 0.013; *I*^2^ = 63.59%, indicates heterogeneity between studies. Visual inspection of a funnel plot does not lead to the assumption of publication bias (Fig. 7).

### Language abilities

**Fig. 4:**
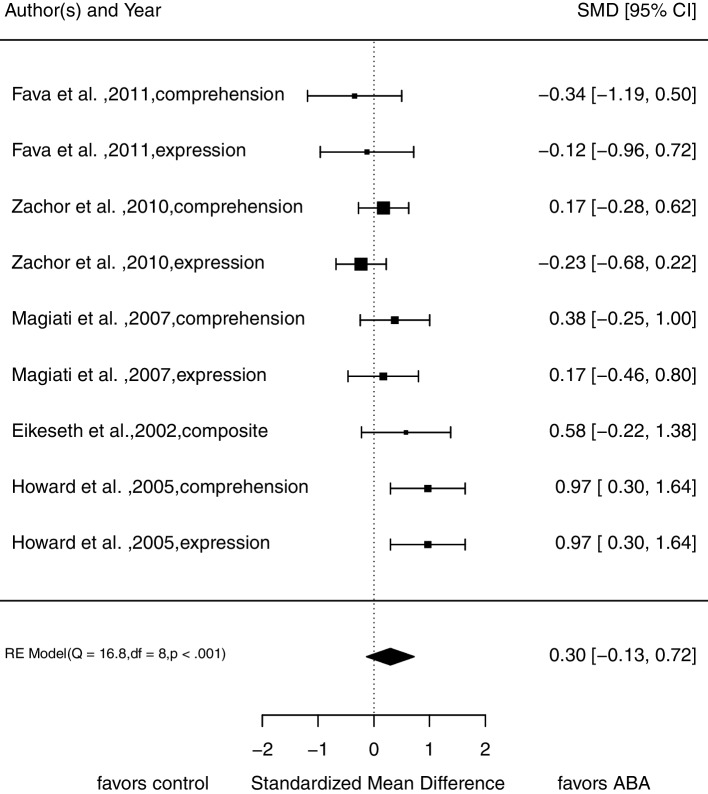
Forrest plot of pooled *SMD* in post-treatment scores in language abilities

We did not find a significant difference in post-treatment scores between treatment and control group regarding language abilities (*SMD* = 0.30, 95% CI [-0.13; 0.72]; Fig. 4). We included five studies from which we calculated nine effect sizes (*N* = 210 at pre-treatment assessment). Analyses show no variance between the dimensions of language abilities (expression and comprehension; ${\upsigma }_{W}^{2}$ = 0.00). Again, there is substantial variance between studies (${\upsigma }_{B}^{2}$ = 0.17) as well as substantial heterogeneity *Q*(8) = 16.81, *p* = 0.03; *I*^*2*^ = 61.94%. Visual inspection of the funnel plot indicates no funnel plot asymmetry (see Fig. 7).

### Symptom severity

**Fig. 5:**
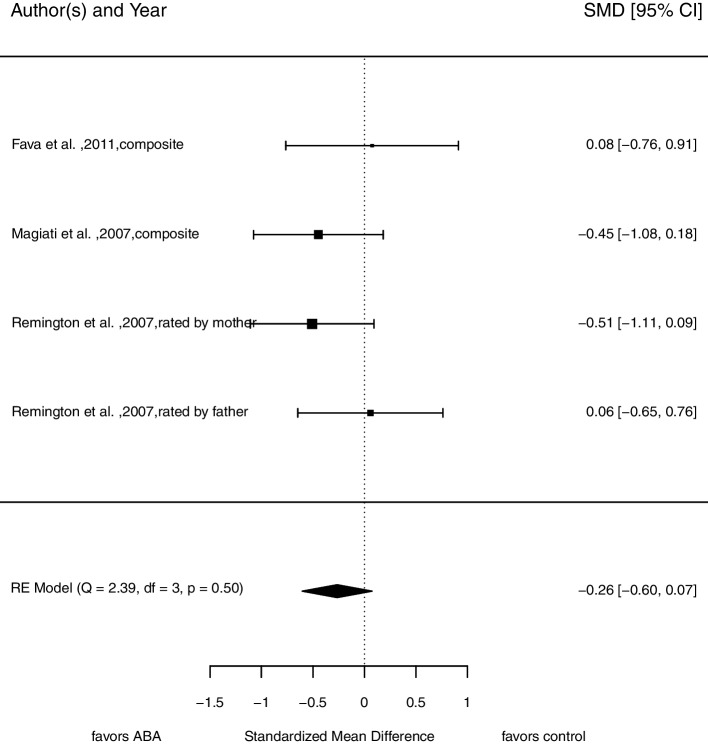
Forrest plot of pooled *SMD *in post-treatment scores in symptom severity

There is no strong evidence for lower levels of symptom severity in children receiving comprehensive ABA-based treatments compared to children in other treatment conditions after intervention (*SMD* = -0.26, 95% CI [-0.60, 0.07]; see Fig. 5). This analysis is based on four comparisons from three different studies with 107 participants at pre-treatment assessment. There is neither variance between dimensions (mother’s and father’s rating of child’s symptoms; ${\upsigma }_{B}^{2}$ = 0.00) nor between studies (${\upsigma }_{B}^{2}$ = 0.00). Based on the *Q* statistic, we assume no substantial heterogeneity, *Q*(3) = 2.39, *p* = 0.50; *I*^2^ = 0.00%. We found no evidence for funnel plot asymmetry (see Fig. 7), but the low number of studies limits a proper interpretation of the funnel plot.

### Parental stress

**Fig. 6:**
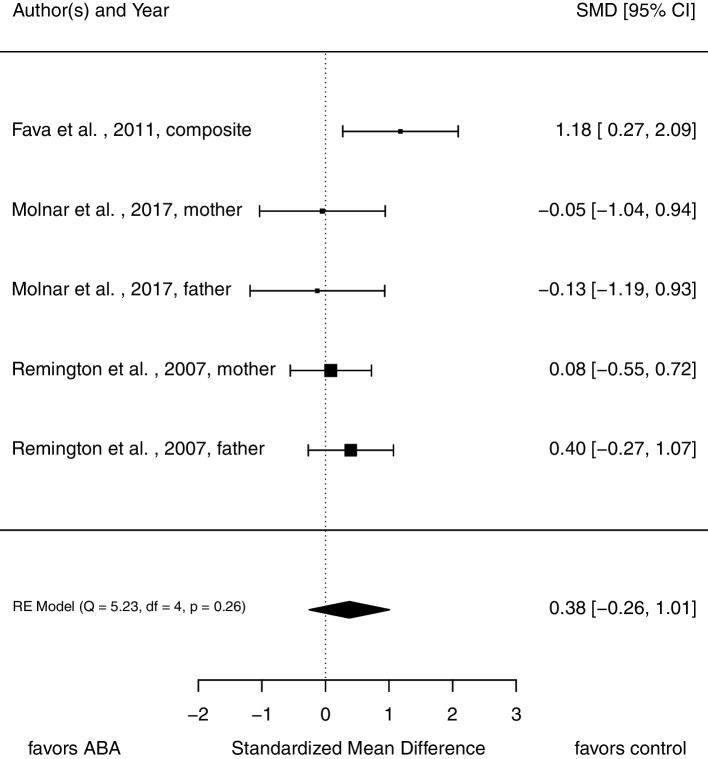
Forrest plot of pooled *SMD* in post-treatment scores in parental stress

For this analysis, five comparisons from three studies with 128 participants at pre-treatment assessment were included. As Fig. 6 displays, there is no evidence for a substantial difference in stress ratings between parents in each intervention condition (*SMD* = 0.38, 95% CI [-0.26, 1.01]).

**Fig. 7:**
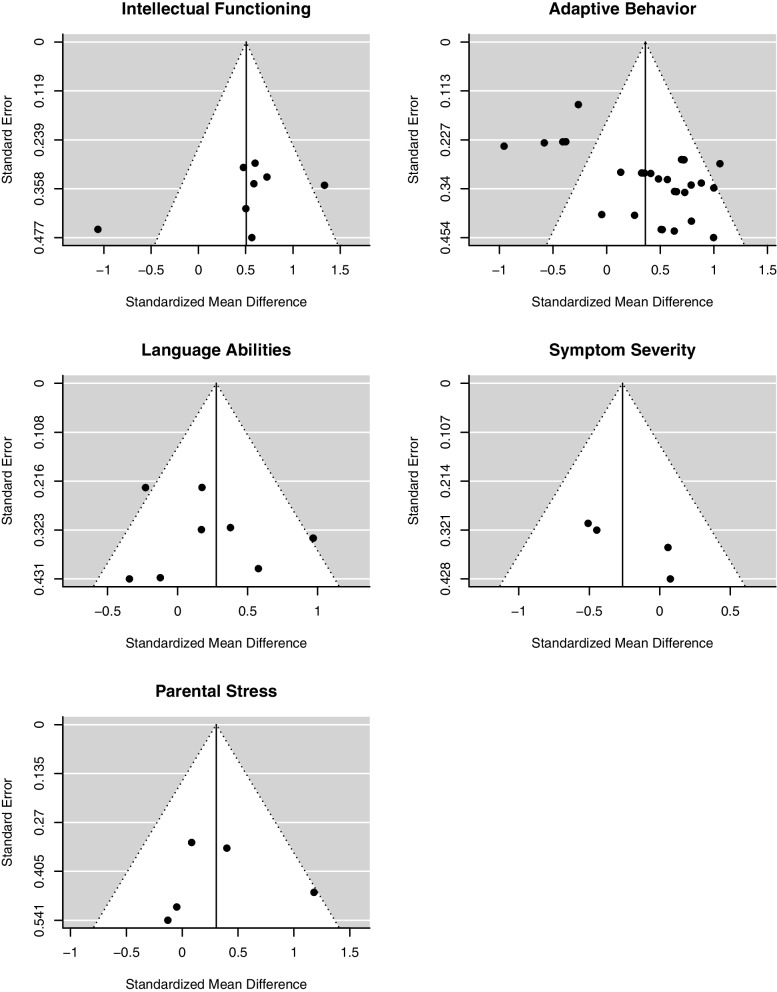
Funnel plots used for publication bias assessment

There is no variance between dimensions of the construct (mother’s and father’s stress rating; ${\upsigma }_{W}^{2}$ = 0.00) but substantial variance between studies (${\upsigma }_{B}^{2}$ = 0.19). The *Q* statistic indicates no heterogeneity, *Q*(4) = 5.23, *p* = 0.26. The calculation of *I*^2^ resulted in *I*^2^ = 52.24%. The funnel plot does not hint toward publication bias (see Fig. 7). Again, validity of this plot is limited, because of the low number of included studies.

### Moderator analyses

Table 4 shows the results of moderator analyses. Due to the small number of eligible studies, we were not able to conduct all planned moderator analyses. We found a significant influence of language abilities (comprehension) at intake on the effect size in intellectual functioning and adaptive behavior. Further, the moderator analyses indicate that abilities of language expression at intake influence the effect size in intellectual functioning and language abilities. Additionally, we inspected possible interactions between treatment duration, intensity as well as total amount of time spent in comprehensive ABA-based interventions and age. Evaluating tests of moderators on the basis of *Q* statistics indicate an interaction between age and treatment intensity for adaptive behavior, which indicates that the influence of treatment intensity on post-treatment adaptive behavior decreases with older age (*β* = *-*0.01, [-0.01, -0.00], *Q*_M_(3) = 74.45, *p* < 0.001). The validity of all described examinations is restricted due to the small number of eligible studies. Therefore, results should have no more than an indicative value.

**Table 4:** Estimated influence of treatment and children characteristics on the post-treatment comparison effect size (β [95%CI])

Potential moderators			Intellectual functioning	Adaptive behavior	Language abilities	Symptom severity	Parental stress
Characteristics of	children	Age	-0.01 [-0.05, 0.02] *Q*_M_(1) = 0.58, *p* = .44	0.02 [-0.01, 0.05] *Q*_M_(1) = 1.44, *p* = .23	0.00 [-0.03, 0.04] *Q*_M_(1) = 0.03, *p* = .85	0.02 [-0.06, 0.12] *Q*_M_(1) = 0.42, *p* = .52	-0.01 [-0.13, 0.11] *Q*_M_(1) = 0.03, *p* = .87
		Intake IF	0.04 [-0.05, 0.06] *Q*_M_(1) = 0.02, *p* = .90	-0.02 [-0.06, 0.03] *Q*_M_(1) = 0.56, *p* = .45	-0.02 [-0.07, 0.03] *Q*_M_(1) = 0.41, *p* = .52	-0.01 [- 0.05, 0.02] *Q*_M_(1) = 0.48, *p* = .49	-
		Intake AB	0.04 [-0.13, 0.21] *Q*_M_(1) = 0.23, *p* = .63	-0.01 [-0.10, 0.08] *Q*_M_(1) = 0.05, *p* = .83	0.02 [-0.08, 0.12] *Q*_M_(1) = 0.17, *p* = .68	-	-
		Intake L (exp.)	0.11 [0.03, 0.19] *Q*_M_(1) = 7.00, *p* = .008	0.04 [-0.02, 0.09] *Q*_M_(1) = 1.42, *p* = .23	0.04 [0.02, 0.07] *Q*_M_(1) = 10.30, *p* = .001	-	-
		Intake L (comp.)	0.60 [0.26, 0.96] *Q*_M_(1) = 11.98, *p* < .001	0.07 [0.03, 0.11] *Q*_M_(1) = 14.69, *p* < .001	0.04 [-0.03, 0.11] *Q*_M_(1) = 1.04, *p* = .31	-	-
		Duration	0.04 [-0.03, 0.10] *Q*_M_(1) = 1.25, *p* = .26	0.00 [-0.05, 0.06] *Q*_M_(1) = 0.00, *p* = .97	0.02 [-0.06, 0.09] *Q*_M_(1) = 0.26, *p* = .61	-	-0.03 [-0.15, 0.09] *Q*_M_(1) = 0.28, *p* = .60
		Intensity	0.04 [-0.02, 0.09] *Q*_M_(1) = 1.72, *p* = .19	0.00 [-0.06, 0.06] *Q*_M_(1) = 0.00, *p* = .99	0.03 [-0.01, 0.06] *Q*_M_(1) = 2.82, *p* = .09	-0.02 [-0.09, 0.03] *Q*_M_(1) = 0.95, *p* = .33	-0.05 [-0.23, 0.12] *Q*_M_(1) = 0.36, *p* = .54
		Intensity (cumulative)	0.00 [-0.00, 0.00] *Q*_M_(1) = 2.27, *p* = .13	0.00 [-0.0, 0.00] *Q*_M_(1) = 0.11, *p* = .74	0.00 [-0.00, 0.00] *Q*_M_(1) = 1.76, *p* = .18	-0.00 [-0.00, 0.00] *Q*_M_(1) = 0.94, *p* = .33	-0.00 [-0.00, 0.00] *Q*_M_(1) = 0.21, *p* = .65

Note. *IF* Intellectual functioning, *AB* Adaptive behavior, *L* Language abilities, *exp* expression, *comp* comprehension

Table 5 displays that some variables considered as moderators are highly correlated. Therefore, it would be necessary to conduct moderator analyses controlling for the influence of other potential moderators. The low number of included studies prevents such analyses. Still, the high correlations have to be kept in mind when drawing conclusions from those moderator analyses.

**Table 5:** Correlations between potentially moderating variables

Variable	Age	Intake IF	Intake AB	Intake L (exp.)	Intake L (comp.)	Duration	Intensity	Intensity (cumulative)
Age	1							
Intake IF	-0.33	1						
Intake AB	-0.67	-0.17	1					
Intake L (exp.)	0.57	-0.77	-0.28	1				
Intake L (comp.)	0.70	-0.98	-0.30	0.82	1			
Duration	-0.24	0.05	-0.10	0.53	-0.02	1		
Intensity	0.04	0.64	-0.51	0.81	0.28	0.36	1	
Intensity (cumulative)	-0.19	0.46	-0.41	0.67	0.08	0.84	0.77	1

*Note*. *IF *Intellectual functioning, *AB*  Adaptive behavior, *L*  Language abilities, *exp* expression, *comp*. comprehension

## Discussion

The current meta-analysis investigated the effect of comprehensive, intensive interventions based on Applied Behavior Analysis (ABA) for ASD versus treatment as usual (TAU), minimal or no treatment on adaptive behavior, intellectual functioning, language abilities, symptom severity, and parental stress. Additionally, the current meta-analysis focused on the potential moderating influence of treatment and demographic characteristics while updating previous meta-analyses and overcoming their methodological limitations. Most studies (with the exception of Shawler [48] and Molnár and Eldevik [55]) included in our meta-analysis were also included in previous meta-analysis, however, no meta-analysis focused on all eleven studies. Our meta-analysis revealed that most studies that were eligible to be included in this review were of low methodological quality. Therefore, the results of our meta-analysis might be affected by the high risk of bias in the included studies. We discuss this issue in greater detail in the limitations section of our discussion.

The results based on post-treatment comparison effect sizes reveal that comprehensive ABA-based interventions (compared to TAU, minimal or no treatment) have a medium effect on intellectual functioning (8 effect sizes) and small effect on adaptive behavior (28 effect sizes), according to Cohen’s conventions [77]. Thus, children who receive comprehensive ABA-based treatments tend to show stronger improvements in intellectual functioning and adaptive behavior than children receiving TAU, minimal or no treatment, which is in line with previous meta-analyses (e.g., [8, 12, 14]). Our results did not indicate differences between treatment and control group in post-treatment scores for language abilities, symptom severity or parental stress. The current analyses revealed overall smaller effect sizes than most previous meta-analyses (e.g., [8, 16–18]). This is most probable caused by the more conservative inclusion criteria. In contrast, Sandbank et al. [12] reported a smaller effect size (estimated summary effects) for behavioral interventions on intellectual functioning (effect size = 0.29* vs. 0.51*, study *N* = 21 vs. 9) and a similar effect size for adaptive behavior (effect size = 0.38* vs. 0.37*, study *N* = 21 vs. 9). However, in their comprehensive study on seven different types of early interventions for ASD, they applied a broader definition of behavioral interventions and assigned more studies to this intervention type, accordingly (besides EIBI, the Lovaas Model, and Verbal Behavior also studies on PECS, Discrete Trial Training, and Autism Partnership). Opposed to Virués-Ortega [8] and Reichow and Wolery [20], we found no evidence for publication bias for intellectual functioning and language abilities. However, our analyses indicated publication bias for adaptive behavior.

Five studies were included to investigate the effects on symptom severity and parental stress. The analyses also did not reveal effects of comprehensive ABA-based treatments versus control group treatments indicating that none of the treatments are superior to each other in reducing symptom severity and parental stress. The lack of evidence for a reduction in symptom severity due to comprehensive ABA-based interventions beyond the effect of other treatments found in this study is in line with findings in the updated review of Reichow and colleagues [14] but different from Sandbank et al.’s [12] findings, who reported an effect size of 0.45 [0.26; 0.68].

To our knowledge, no other meta-analysis besides our own has calculated an effect size for parental stress yet. Even though Schwichtenberg and Poehlmann [37] found that parental involvement in comprehensive ABA-based interventions increased maternal strain, we did not find evidence for impact of comprehensive ABA-based interventions on parental stress beyond the impact of treatment as usual, no or minimum treatment. However, because of the small number of studies included in these analyses a negative or non-detected positive effect for parental stress and symptom severity has to be considered. Therefore, a definite conclusion would be premature.

As outlined above, previous research regarding moderating variables in comprehensive ABA-based treatments is inconsistent. Because of several aspects discussed in the limitation section, results of moderator analyses in this study have to be interpreted with caution. The current moderator analyses indicated that higher language abilities at intake are beneficial for gains in intellectual functioning and adaptive behavior. This finding supports findings of previous primary studies (e.g., [31]) and meta-analyses (e.g., [27]). The interaction between age and treatment intensity for adaptive behavior indicates a decreasing influence of treatment intensity on adaptive behavior with older age. A replication of our results is needed, before sound conclusions can be drawn. However, if the finding on a decreased influence of treatment intensity on adaptive behavior with older age replicated, this might inform decisions on treatment indications.

### Limitations

Primary studies are the basis of every meta-analysis. The studies included in the present meta-analysis investigated children with a mean age between 2.2 and 5.5 years at intake, mostly without comorbidities and mostly from western countries, limiting generalization of results to these demographic characteristics. Another source of bias could be parents’ acceptance of treatment, since group assignment was based on parental preference in most studies. Besides limitations regarding participant characteristics, included primary studies and, thus, this meta-analysis are limited by a very low quality of evidence determined with the approach from Higgins and Green [23]. Concerns regarding the influence of selection and performance biases are raised by the facts that no study reported an adequate randomization procedure, allocation was not concealed, and personnel and participants were not masked. However, not masking personnel and participants is a limitation that can hardly be overcome in future studies, since it is incompatible with training parents and personnel to apply intensive ABA-based treatments. Another source of bias lies within the control groups, which are only vaguely defined and varied a lot in their intensity (*M* = 17.19 h/week, *SD* = 10.83). Thus, they are hardly comparable and prone to contamination. For instance, if TAU groups applied ABA-based methods to a large extent (e.g., 9 h per week) without reporting this clearly, the effect size for comprehensive, intensive ABA-based interventions could be underestimated. Future studies should precisely report which interventions were delivered to which extent. Several studies reported baseline imbalances between groups, which indicates possible confounding variables. Furthermore, some outcome measures, for example VABS or ASQ, are based on parental reporting. These reports might be biased since group assignment was based on parental preference. The low quality of evidence appears to be a common problem within the research on treatment for autistic children (see [14] and [12]). This limits the informative value of meta-analyses in this field. Therefore, Sandbank and colleagues [12] recommend several approaches that could help to increase the methodological quality of studies with autistic children. For example, they recommend that outcomes are assessed by trained assessors and not parents or teachers. We support the statement of Sandbank and colleagues that researchers should continue to strive to conduct high-quality studies. Another starting point to improve methodological quality, is the assessment of intellectual functioning (a core developmental outcome). Most studies used instruments measuring IQ with verbal tasks [78]. Therefore, better scoring in those instruments might also reflect improved language abilities. Additionally, several studies used more than one instrument and calculated average IQ scores over all instruments. This would only be legitimate, if all used instruments measured the exact same construct — an assumption none of the studies validated. It can be difficult to find one instrument that is valid for all study participants, as they vary regarding age and intellectual impairment. However, we suggest that future studies may use as few instruments as possible to ensure comparability between participants. Finally, it has to be considered, that young autistic children often underperform in tests on intellectual functioning, for example, due to motivational reasons. Another limitation is, that some relevant studies could not be included because required data were not reported and could not be obtained by the authors.

An important limitation of our study is the effect size selection. As described above, it is recommended by the Cochrane Handbook [23] to use post-treatment comparisons, if standard deviations for change scores are not reported in primary studies. However, it is also stated that this procedure should be unproblematic for randomized trials. It should be noted, that effect sizes based on post-treatment comparisons could be biased due to baseline differences in treatment and control group if most primary studies applied a quasi-experimental design. The results of the moderator analyses may be interpreted as preliminary, as the number of primary studies including these moderators was low. Especially symptom severity at intake was assessed rarely and with different measures in primary studies. Most measures were not originally designed to quantify symptom severity, even though higher scores in ADI-R and ADOS indicate more deficits [74, 79]. The guidelines for the diagnostics of ASD from the Association of the Scientific Medical Societies (AMWF) recommend to use the ADI-R and the ADOS in order to support the diagnostic process in children with ASD (for the detailed recommendation see [80]). If future studies endorse this recommendation, they should report the results of those assessments. Nevertheless, the development of a reliable instrument for the assessment of symptom severity in children with ASD would be preferable. Furthermore, the number of studies including parental stress was too low to conduct moderator analyses. To sum up, results from our moderator analyses, especially the examination of interactions between moderators, are merely indicative at the time being and have to be replicated. Additionally, further and more complex analyses have to be conducted, for example to account for the correlation between investigated variables. Therefore, we conclude that moderators and their interactions cannot be investigated properly until more studies contribute to the analysis.

This study excluded single case experimental design studies, even though they are commonly administered in the research on treatments for autistic children (e.g., [12]). However, while we think that single case designs are important, since there is a large heterogeneity in autistic individuals, which is better addressed in single case studies, we also agree with Sandbank and colleagues [12], that studies with a controlled design are needed to explore the generalizability of the effects of comprehensive ABA-based interventions. Next to the concerns expressed in Sandbank et al. [12], we believe that the inclusion of single case studies in our meta-analysis would have added to the already high heterogeneity in studies included in our meta-analysis, thus further limiting the conclusiveness of our results.

Importantly, besides the understanding of ASD as a neurodevelopmental disorder, as can be found in the ICD or DSM, autism is also conceptualized in a neurodiversity framework by many autistic individuals, researchers and clinicians, namely as a natural variation of neurological diversity [e.g., 81]. It is being discussed whether early interventions in general are at all compatible in this framework, as they are usually focused on curing or reducing impairments rather than on the strengths associated with neurodiversity [e.g., 82, 83]. An in-depth discussion of the conceptualization of autism as well as ethical considerations regarding comprehensive ABA-based interventions in children with ASD is limited in this meta-analysis because it is beyond the scope. Nevertheless, 1) addressing concerns which are held against comprehensive ABA-based treatments and investigating undesirable side effects in behavioral treatments, as recommended by the National Institute for Health and Care Excellence [84], as well as 2) considering changes in comprehensive ABA-based interventions acknowledging the neurodiversity framework appear important to improve the support for autistic children and their families.

## Conclusions

Several meta-analyses, including the current study, revealed evidence for a medium effect of comprehensive ABA-based interventions (vs. treatment as usual, minimal or no treatment) on intellectual functioning and adaptive behavior. However, the current meta-analysis did not revealed support for effects on language abilities, symptom severity, and parental stress beyond the control group treatments. Methodological limitations of primary studies and, thus, this meta-analysis may bias these results (e.g., low number of studies for analysis on symptom severity). However, the effect of comprehensive ABA-based interventions on core features of ASD may be comparable to the effects of the control group treatments. As comprehensive ABA-based interventions investigated in this meta-analysis contained more treatment hours (ABA 21.85 h/week, *SD* = 5.90 vs. CG 17.19 h/week, *SD* = 10.82), one may conclude that they are overly extensive and not justified. But then, if comprehensive ABA-based treatments (compared to no or minimal treatment or TAU) lead to higher levels of adaptive behavior and intellectual functioning as our study indicates, they would decrease the differences between the developmental and actual age of children on the autistic spectrum in these areas. Improvements of this kind can make major differences in the daily life of the children and their families.

Still, to answer the question, whether comprehensive ABA-based interventions are valid treatments for ASD, to full extent, more methodological sound studies are needed. Thus, robust conclusions on the effectiveness are still limited by a low number and rather low quality of primary studies. This applies also to the moderating influence of treatment characteristics, such as treatment intensity, and child characteristics, such as age. Conclusive knowledge in regard to effectiveness and moderators would help professionals to decide about indication of different treatment options and would help parents of children with ASD to make an informed decision. Other statistical approaches, e.g. growth curve analyses as seen in the study by Tiura and colleagues [31], might help to develop personalized treatment options [85]. Additionally, future research may aim to overcome limitations of previous studies. For example, the (further) development and evaluation of diagnostic procedures in children with ASD is sorely needed [80]. This would lower the risk for biases in meta-analytic methods. Further, the ethical concerns for RCTs on ASD treatment, namely the concern that critical developmental stages of the children might pass during the treatment in potentially non-profitable treatment groups, may be addressed by either comparing two potentially helpful interventions [78] or using new methodological approaches, such as adaptive rolling designs [86].

## Supplementary Information

**Additional file 1**.

## Data Availability

The datasets generated and/or analyzed during the current study are available in the OSF repository, https://osf.io/wfgu3/.

## Abbreviations

ABA: Applied Behavior Analysis
ABA-VB: Applied Behavior Analysis—Verbal Behavior Approach
ADI-R: Autism Diagnostic Interview – Revised
ADOS: Autism Diagnostic Observation Schedule
AMWF: Association of the Scientific Medical Societies
APA: American Psychiatric Association
ASD: Autism Spectrum Disorder
ASQ: Autism Screening Questionnaire
BSID: Bayley Scales of Infant Development
CI: Confidence Interval
DSM: Diagnostic and Statistical Manual of Mental Disorders
EIBI: Early Intensive Behavioral Interventions
ESDM: Early Start Denver Model
ICD: International Statistical Classification of Diseases and Related Health Problems
NDBI: Naturalistic Developmental Behavioral Interventions
NICE: National Institute for Health and Care Excellence
PECS: Picture Exchange Communication System
PSI: Parenting Stress Index
QRS: Questionnaire on Resources and Stress–Friedrich Short Form
RCT: Randomized Controlled Trial
RDLS: Reynell Developmental Language Scale
SB:FE: Stanford-Binet Intelligence Scale: Fourth Edition
SMD: Standardized Mean Difference
TAU: Treatment as Usual
TEACCH: Treatment and Education of Autistic and Related Communication Handicapped Children
VABS: Vineland Adaptive Behavior Scale
WISC: Wechsler Intelligence Scale for Children
WPSSI: Wechsler Preschool and Primary Scale of Intelligence

## Footnotes

1 In line with Virués-Ortega [8], we chose to set the limit for intensity lower than it should be according to the definition of ABA-based treatments. Thus, we were able to achieve more variety in treatment intensity. This was necessary to conduct a moderator analysis considering treatment intensity.
