# Supplementary material for: Comprehensive ABA-based interventions in the treatment of children with autism spectrum disorder – a meta-analysis
Source: BMC Psychiatry. 2023 Mar 2;23:133. doi: 10.1186/s12888-022-04412-1 (PMC9983163; doi:10.1186/s12888-022-04412-1)
Supplement: Supplementary file 1 — Additional file 1. [file 12888_2022_4412_MOESM1_ESM.docx]

Supplementary Material

**Supplementary Material A: Supplementary Material for Literature Search and Data Extraction**

**Search Strategy**

(ASD OR autism OR autism spectrum disorder OR Autismus OR ASS OR Autismus-Spektrum-Störung) AND (EIBI OR ABA OR „early intensive behavio* intervention*“ OR „applied behavio* analysis“ OR „comprehensive aba“ OR „early intensive behavio* treatment“ OR „ucla model“ OR Lovaas OR "intensive behavio* training" OR "pivotal response training" OR Vehaltensanalyse OR Frühförderung OR "early start denver model" OR "Autismusspezifische Verhaltenstherapie" OR "Angewandte Verhaltenstherapie")

**Data Collection Form**

The data collection form was created using Microsoft Excel. The following list contains all considered variables (and a short explanation). The original data collection form is not displayed due to a lack of space. But it is attached in the electronic supplement.

- Study ID (each study was identified by a number)
- Report ID (each report was identified by a number. If two reports refer to the same study, the same number was assigned)
- Rater ID (each rater was identified by a number)
- Eligibility (is the study eligible? 0 = no, 1 = yes)
- Reasons for exclusion
- Author(s)
- Year of publication
- Country (in which the study was conducted)
- Study design
- Number of intervention groups
- ABA Model
- Trainer (who trained the child? 0 = therapists, 1 = parents, 2 = both)
- Training (of personnel and parents)
- Control group (what kind of control group was used)
- Sample Size
- Sample size TG (sample size treatment group)
- Sample size CG (sample size control group)
- Mean age in months
- Mean age TG (in months; with range and standard deviation)
- Mean age CG (in months; with range and standard deviation)
- Setting (in which setting was the treatment delivered? 0 = clinical/center-based, 1 = at school, 2 = home-based, 3 = multiple setting)
- Male (%) (Proportion of male participants)
- Instrument used to make diagnosis (e.g., ADI-R, ADOS)
- Co-morbidity (Which co-morbidities were mentioned? How many children have co-morbid conditions?)
- Mean intensity TG (in h/week; with range and standard deviation)
- Mean intensity CG (in h/week; with range and standard deviation)
- Mean duration TG (in months; with range and standard deviation)
- Mean duration CG (in months; with range and standard deviation)
- Intake intellectual functioning (average level of intellectual functioning at intake)
- Intake adaptive behavior (average level of adaptive behavior at intake)
- Intake language comprehension (average level of language comprehension at intake)
- Intake language expression (average level of language expression at intake)
- Intake symptom severity (average level of symptom severity at intake)
- Instrument used to measure intellectual functioning
- Instrument used to measure adaptive behavior
- Instrument used to measure language abilities
- Instrument used to measure symptom severity
- Instrument used to measure parental stress
- control group ID (if a study had more than one control group, each group was identified by a number)
- For each outcome/outcome dimension the following was coded:
  - Scale (which scale was reported? E.g., standard score or raw score?)
  - Mean TG pre
  - Standard deviation TG pre
  - Sample size TG pre
  - Mean CG pre
  - Standard deviation CG pre
  - Sample size CG pre
  - Mean TG post
  - Standard deviation TG post
  - Sample size TG post
  - Mean CG post
  - Standard deviation CG post
  - Sample size CG post

**Supplementary Material B: Inter-rater Agreement for all Potential Moderator and Outcome Variables**

Inter-rater Agreement for all Potential Moderator and Outcome Variables prior and after discussion

| Variable | ICC(2,1)_pre_ | ICC(2,1)_post_ |
| --- | --- | --- |
| Eligibility | .4* | 1* |
| Age | 1 | 1 |
| Intake adaptive behavior | NA | 1 |
| Intake intellectual functioning | NA | 1 |
| Intake language comprehension | NA | 1 |
| Intake language expression | NA | 1 |
| Intake symptom severity | NA | 1 |
| Trainer | .74* | .79* |
| Duration | .999 | 1 |
| Intensity | .575 | 1 |
| Mean TG pre | 1 | 1 |
| Standard deviation TG pre | 1 | .999 |
| Sample size TG pre | 1 | 1 |
| Mean CG pre | .999 | 1 |
| Standard deviation CG pre | .988 | .993 |
| Sample size CG pre | 1 | .999 |
| Mean TG post | .999 | 1 |
| Standard deviation TG post | .995 | .996 |
| Sample size TG post | 1 | 1 |
| Mean CG post | 1 | 1 |
| Standard deviation CG post | .767 | .802 |
| Sample size CG post | 1 | 1 |

Note. ICC(2,1)_pre_ = inter-rater agreement for 9 studies prior discussion and reassessment, ICC(2,1)_post_ = inter-rater agreement for 9 studies after discussion and reassessment. Values marked with asterisk (*) do not represent ICC(2,1) but Cohen’s kappa (κ). Values represent absolute agreement after reassessment of studies.

**Supplementary Material C: Brief description of the “Cochrane Collaboration’s tool for assessing risk of bias” by Higgins & Green, 2011**

The “Cochrane Collaboration’s tool for assessing risk of bias” (Higgins & Green, 2011) appraises following quality features: (a) assignment of participants to different conditions is based on chance (*sequence generation*); (b) participants’ eligibility and their informed consent are independent from the upcoming assignment to a condition (*allocation sequence concealment*); (c) participants and personnel are not aware of the treatment condition the participant is in (*blinding of participants and personnel*); (d) people who assess outcome measurements do not know the treatment condition a participant is in (*blinding of outcome assessors*); (e) the study provides complete outcome data or addresses missing data adequately, for example uses appropriate imputation strategies (*incomplete outcome data*); (f) the study does not withhold data systematically, for example non-significant results (*selective outcome reporting*); (g) the study is free from other risks of bias, e.g. contamination or baseline imbalance (*other risk of bias*). Each feature was evaluated and labeled with “low risk”, “unclear risk” or “high risk” for all studies included.

Supplementary Material D: List of all outcome measures

| Outcome | Instrument | Version | Author, Year |
| --- | --- | --- | --- |
| Adaptive  Behavior | Denver Developmental Screening Test II | DDST-II | Frankenburg, Dodds, Archer, Shapiro, & Bresnick, 1992 |
|  | Developmental Profile | DP-II | Alpern, Boll, & Shearer, 1986 |
|  | Repetitive Behavior Scale | RBS | Bodfish, Symons, &, Lewis, 1998 |
|  | Rockford Infant Development Evaluation Scales | RIDES | Project RHISE, 1979 |
|  | Vineland Adaptive Behavior Scale | VABS I, VABS II | Sparrow, Balla, &, Cicchetti, 1984, 2005 |
| Intellectual Functioning | Bayley Scales of Infant Development | 2nd and 3rd Edition | Bayley, 1993, 2006 |
|  | Development Assessment of Young Children | DAYC | Voress & Maddox, 1998 |
|  | Developmental Profile | DP-II | Alpern, Boll, & Shearer, 1986 |
|  | Differential Abilities Scale | DAS | Elliott, 1990 |
|  | Griffith mental developmental Scales | GMDS-ER 2-8, GMSD | Luiz, Barnard, Knosen, Kotras, Horrocks, McAlinden, et al., 2006, Alin-Åkerman & Nordberg, 1980 |
|  | Mullen Scales of Early Learning | MSEL | Mullen, 1995, 1997 |
|  | Psychoeducational Profile | PEP-R, PEP-3 | Schopler, Reichler, Bashford, Lansing, & Marcus, 1990; Schopler, Lansing, Reichler, & Marcus, 2005 |
|  | Stanford-Binet Intelligence Scale: | 4th and 5th Edition | Thorndike, Hagen, & Sattler, 1986, Roid, 2003 |
|  | Wechsler Intelligence Scale for Children | WISC-R, WISC-III | Wechsler, 1974, 1996 |
|  | Wechsler Preschool and Primary Scale of Intelligence | WPPSI-R, WPPSI-III, WPPI-R | Wechsler, 1989, Wechsler, 2005 |
| Language | British Picture Vocabulary Scale- II | BPVS-II | Dunn, Dunn, Whetton, & Burley, 1997 |
|  | Expressive One-Word Picture Vocabulary Test | EOWPVT, EOWPVT-R | Brownell, 2000a, Gardner, 1990 |
|  | Expressive Vocabulary Test | EVT | Williams, 1997 |
|  | Infant-Toddler Developmental Assessment |  | Provence, Eriksen, Vater, & Palmeri, 1985 |
| Appendix D (continued) | |  |  |
| Outcome | Instrument | Version | Author, Year |
| Language | MacArthur Communication Developmental Inventories | CDI | Fenson, Pethick, Renda, & Cox, 2000 |
|  | Peabody Picture Vocabulary Test-3rd Edition | PPVT-III | Dunn & Dunn, 1997 |
|  | Preschool Language Scale-3 | PLS-3 | Zimmerman, Steiner, & Pond, 1992 |
|  | Receptive-Expressive Emergent Language Scale-Revised | REEL-2 | Bzoch & League, 1991 |
|  | Receptive One-Word Picture Vocabulary Test | ROWPVT | Brownell, 2000b |
|  | Reynell Developmental Language Scales | RDLS, RDLS III | Reynell & Gruber, 1990, Edwards, Fletcher, Garman, Hughes, Letts, & Sinka., 1997 |
|  | Rossetti Infant-Toddler Language Scale |  | Rossetti, 1990 |
|  | Sequenced Inventory of Communication Development-Revised Edition | SICD-R | Hedrick, Prather, & Tobin, 1984 |
|  | Language scales of WISC-R, WPPSI-R & DP-II |  |  |
| Symptom  severity | Autism Diagnostic Interview – Revised | ADI-R | Lord, Rutter & Le Couteur, 1994 |
|  | Autism Diagnostic Observation Schedule | ADOS | Lord, Rutter, DiLavore, & Risi, 1999, 2002 |
|  | Autism Screening Questionnaire | ASQ | Berument, Rutter, Lord, Pickles, & Bailey, 1999 |
|  | Childhood Autism Rating Scale | CARS,  CARS2-ST, CARS2-HF | Schopler. Reichler, & Renner, 1986, Schopler, Reichler, & Rochen Renner, 2010, Schopler, Van Bourgondien, Wellman, & Love, 2010 |
| Parental Stress | Parenting Stress Index | PSI | Abidin, 1995 |
|  | Questionnaire on Resources and Stress–Friedrich short form | QRS | Friedrich, Greenberg, & Crnic, 1983 |
